# A Bayesian Prediction Spatial Model for Confirmed Dengue Cases in the State of Chiapas, Mexico

**DOI:** 10.1155/2022/1971786

**Published:** 2022-05-25

**Authors:** Manuel Solís-Navarro, Cruz Vargas-De-León, María Gúzman-Martínez, Josselin Corzo-Gómez

**Affiliations:** ^1^Sección de Estudios de Posgrado, Escuela Superior de Medicina, Instituto Politécnico Nacional, Ciudad de México, Mexico; ^2^Facultad de Matemáticas, UAGro, Ciudad Universitaria s/n Chilpancingo, Chilpancingo, Guerrero, Mexico; ^3^División de Investigación, Hospital Juárez de México, Ciudad de México, Mexico; ^4^Escuela de Ciencias Químicas Sede Ocozocoautla de Espinosa, Universidad Autónoma de Chiapas, Tuxtla Gutiérrez, Mexico

## Abstract

Dengue is one of the major health problems in the state of Chiapas. Consequently, spatial information on the distribution of the disease can optimize directed control strategies. Therefore, this study aimed to develop and validate a simple Bayesian prediction spatial model for the state of Chiapas, Mexico. This is an ecological study that uses data from a range of sources. Dengue cases occurred from January to August 2019. The data analysis used the spatial correlation of dengue cases (DCs), which was calculated with the Moran index statistic, and a generalized linear spatial model (GLSM) within a Bayesian framework, which was considered to model the spatial distribution of DCs in the state of Chiapas. We selected the climatological, geographic, and sociodemographic variables related to the study area. A prediction of the model on Chiapas maps was carried out based on the places where the cases were registered. We find a spatial correlation of 0.115 (*p* value=0.001)between neighboring municipalities using the Moran index. The variables that have an effect on the number of confirmed cases of dengue are the maximum temperature (Coef=0.110; 95% CrI: 0.076 − 0.215), rainfall (Coef=0.013; 95% CrI:0.008 − 0.028), and altitude (Coef=0.00045; 95% CrI:0.00002 − 0.00174) of each municipality. The predicting power is notably better in regions that have a greater number of municipalities where DCs are registered. The model shows the importance of considering these variables to prevent future DCs in vulnerable areas.

## 1. Introduction

Dengue is an infectious disease that is caused by the dengue virus (DENV), which belongs to the Flavivirus genus. There are four serotypes, which are called DENV 1 − 4, circulating in tropical and subtropical areas of the world [1, 2]. This disease is transmitted by an arthropod (*Aedes aegypti* mosquito). Because it cannot regulate its temperature, this arthropod depends on the environmental conditions for its feeding and reproduction [3].

According to the World Health Organization (WHO), the incidence of dengue disease has grown dramatically in recent years, presenting high rates of morbidity and mortality [1] mainly in Africa, the Middle East, Asia, the Pacific islands, and America [4]. In 2019 alone, there were approximately 4.2 million cases of dengue [5, 6]. In the same year, the highest dengue registry was reported in the Americas with 3,139,335 cases and 1,538 deaths [1]. The 2019 registries show that the most affected areas were Brazil, Central America, and Mexico. In the same year, 41,505 cases with 191 deaths were reported in Mexico; of these, 2,241 cases and 22 deaths occurred in the state of Chiapas [7].

Due to the distribution of the disease, Chiapas has experienced a very high incidence of cases reported in recent years. Chiapas has been one of the states with the highest incidence of cases nationwide, which has forced the responsible departments for disease control to take more effective measures against dengue [8].

Several factors have been associated with the transmission of the virus, which are divided into microfactors (host factors) and macrofactors (environmental and social factors). The macrofactors have favored the spread of the infection to other regions and have led to an increasing number of cases worldwide [9].

Given the lack of an effective vaccine and specific methods to eradicate the disease, the best way to combat the DENV is through prevention. In particular, the application of effective measures to reduce the reproduction rate of vector mosquitoes is crucial to avoiding major outbreaks or epidemics [10]. Therefore, a good prediction of cases at the state level is important to be able to direct prevention resources more efficiently and to reduce the number of cases, considering climatological and geographic factors [11].

Geographic Information Systems (GISs) are able to handle spatially correlated data and they offer support for decision-making in disease control. Although there are still relatively few studies that approach dengue transmission, they show the spread of the disease and characterize the different geographical areas with the climatological characteristics that are directly related to the vector [12]. In addition, GIS works in conjunction with the statistical models to determine the risk factors that predict future dengue cases (DCs) in endemic zones.

In 2019, Bett et al. [13] analyzed data using a hierarchical spatial Bayesian model, which fitted the data well based on Theil's coefficient of inequality. The authors identified temperatures, altitude, rainfall, and area under urban settlement as significant predictors of dengue incidence in Vietnam.

Akter et al.'s [14] study was carried out in Queensland Australia (2021) and analyzed the spatial variation of dengue incidence in relation to climate variability and socioecological factors in the tropical climate zone. The authors developed univariate Poisson regression models in a Bayesian framework with a conditional autoregressive prior structure. Posterior parameters were estimated using Bayesian Markov Chain Monte Carlo simulation with Gibbs sampling. The authors concluded that some of the climatological and socioecological factors explained much of the heterogeneity of dengue transmission dynamics in that zone.

A Bayesian Poisson spatial regression was used in Thailand in 2019 by Phanitchat et al. [5] to analyze the association between monthly disease incidence and climate variations. A hotspot analysis was used to assess the spatial patterns of dengue incidence. Phanitchat et al. showed that dengue incidence was highly seasonal (rainy season) and positively associated with maximum ambient temperature [5].

Statistical models have been used to predict future DCs in many endemic areas around the world [5]. However, there are no spatial modeling studies to predict DCs in Chiapas. Therefore, it is important to apply these models to one of the states with the highest morbidity rates. Furthermore, the participation of macrofactors in the spread of the dengue virus would be of interest given to the climatological and social conditions in the region. The regions with the greatest transmission of the disease should be identified with the help of data georeferencing and the application of statistical models, in addition to analyzing risk factors that produce DCs and determining their dispersion. This would allow us to enhance the control programs through actions directed against the transmitting vector. In addition, it will help us to understand DCs dispersion and prioritize efforts and resources toward the most vulnerable areas to reduce the high rates of morbidity and mortality. Consequently, this study aims to predict the spatial dispersion of DCs in the state of Chiapas.

This paper studies the generalized linear spatial model (GLSM) to establish the prediction of DCs in the state of Chiapas, Mexico. First, we georeferenced each DC, and we obtain the climatological and nonclimatological data of different databases. Second, we select the model with statistically significant variables and evaluate the spatial correlation. On this basis, we evaluate the structure of the selected model. Finally, we use the selected model to predict DCs in the state of Chiapas, Mexico.

## 2. Methods

This is an ecological study that was performed by collecting data from several sources. The DCs occurred in the state of Chiapas, Mexico, from January to August 2019.

### 2.1. Data Description

#### 2.1.1. Study Area

**Figure 1:**
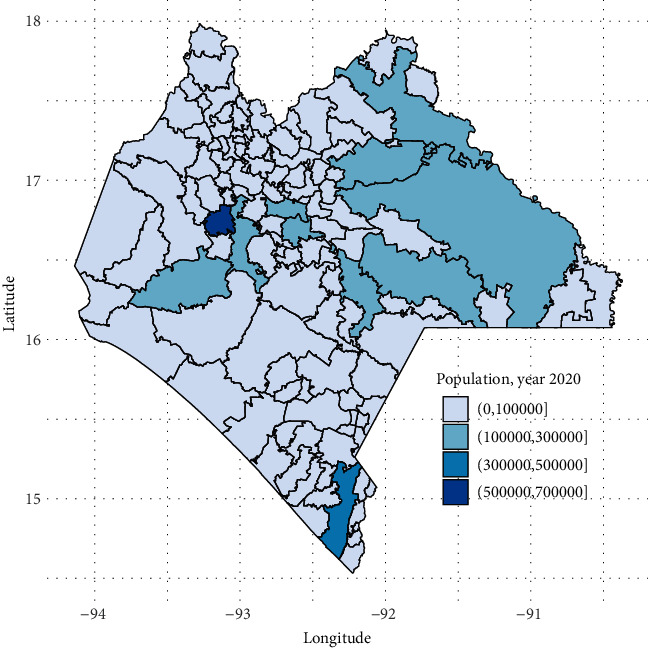
Population distribution of Chiapas.

Dengue is endemic in Chiapas with a scattered record of cases, due to the climatic variability of the state in its different municipalities, also complying with the climatic conditions that favor the reproduction of the vector. Chiapas is divided into 118 municipalities, each with different sociodemographic and climatic conditions. According to the 2020 population and housing census of the National Institute of Statistic and Geography (Spanish acronym: INEGI), the population of the state of Chiapas was 5,543,828. Tuxtla Gutierrez (the capital city) had the highest population density burden with 604,147 inhabitants [15].  Figure 1 shows the population distribution in Chiapas.

#### 2.1.2. Data from Confirmed Dengue Cases

The database of confirmed DCs reported for the state of Chiapas was obtained from the State Health Secretary, in collaboration with the vector-borne diseases area. This database is updated weekly, fulfilling 52 epidemiological weeks (EWs) reports per year. A weekly report is given to the General Directorate of Epidemiology where a previous diagnosis is obtained that shows suspected cases of dengue, which are confirmed by the reverse transcription-polymerase chain reaction (RT-PCR) test in the State Public Health Laboratory, where approximately 10% of the samples are randomly analyzed to obtain a final confirmatory diagnosis.

#### 2.1.3. Nonclimatological Data

External sources such as INEGI were considered to obtain the data on other factors that are related to dengue, such as population density and altitude of each municipality [15]. The following variables were obtained from the original dengue database provided by the State Health Secretary: garbage disposal, contact with the vector, drinking water service, age, and gender.

#### 2.1.4. Climatological Data

The climatological data were obtained from the World Meteorological Organization (WMO) [16], where the maximum temperature and minimum temperature corresponded to the date of infection and the municipality of residence of each of the 573 confirmed cases. The precipitation was added according to the accumulated rainfall during the valuation period and the municipality of residence where DCs were registered in Chiapas [17, 18].

#### 2.1.5. Spatial Location

Georeferencing was carried out using the open-source software R Project for Statistical Computing version 4.0.3 [19]. The packages geoRglm [20–22], ggplot2 [23], and rgdal [24] were used. The World Geodetic System (WGS84) reference coordinate system was used to locate the confirmed cases of dengue from January to August 2019, using the postal code to locate them geographically within a map of Chiapas.

### 2.2. Generalized Linear Spatial Models

Let {*S*(**x**) : **x** ∈ **A** ⊂ *ℝ*^2^} be the Gaussian process that is functionally related to the spatially varying attribute of interest, and **S**=(*S*(**x**_1_),…, *S*(**x**_*n*_))′; then,

(1) $$\begin{array}{c} S∼N_{n}\left(0,\Sigma \right), \end{array}$$

where *E*(*S*(**x**))=**0** and Σ=Cov(*S*(**x**))=*σ*^2^*R*(*ϕ*) is an *n* × *n* positive definite variance-covariance matrix; *σ*^2^ > 0 and *R*(*ϕ*) is the matrix of correlations of the Gaussian process of the dimension *n* × *n* whose elements are given by

(2) $$\begin{array}{c} {\left(R\left(\phi \right)\right)}_{ij}=p\left(h_{ij},\phi \right). \end{array}$$

(*R*(*ϕ*))_*ij*_ is the correlation that exists between *S*(**x**_*i*_) and *S*(**x**_*j*_), *ϕ* is a scale parameter, and *h*_*ij*_=‖**x**_*i*_ − **x**_*j*_‖ is the Euclidean distance that exists between **x**_*i*_ and **x**_*j*_ [25].

Among the parametric functions that exist in the literature for the correlation function *p*(*h*, *ϕ*), where *h*=‖**x** − **x**′‖ is the Euclidean distance and **x**,  **x**′∈*A*, are the following [26]:

Exponential correlation:

(3) $$\begin{array}{c} \rho \left(h,\phi \right)=\text{exp}\left(\frac{-h}{\phi }\right),\;h>0,\;\phi >0. \end{array}$$

Gaussian correlation:

(4) $$\begin{array}{c} \rho \left(h,\phi \right)=\text{exp}\left(-\;{\left(\frac{h}{\phi }\right)}^{2}\right),\;h>0,\;\phi >0. \end{array}$$

Matérn correlation:

(5) $$\begin{array}{c} \rho \left(h,\phi \right)=\frac{1}{2^{\kappa -1}\Gamma \left(\kappa \right)}{\left(\frac{h}{\phi }\right)}^{\kappa }K_{\kappa }\left(\frac{h}{\phi }\right),\;h\geq 0,\;\phi >0,\;\kappa >0, \end{array}$$

where Γ(·) is the gamma function and *K*_*κ*_(·) denotes a modified Bessel function of order *κ*. For *κ*=0.05, the Matérn correlation function (5) reduces to the Exponential correlation function (3).

Given **x**_*i*_,…, **x**_*n*_ *n* spatial locations where the response variable *Y* is observed. At each location *x*_*i*_, the response variable *Y*_*i*_, *i*=1,…, *n*, is associated with a vector of covariates *d*_*j*_(**x**_1_),…, *d*_*j*_(**x**_*n*_), *j*=1,…, *p*. If the random variable *Y*_*i*_ is counted, then generalized linear spatial models (GLSM) [27] can be used.

The model used was

(6) $$\begin{array}{c} Y_{i}|S\left(\cdot \right)∼\text{Poisson}\left(\mu_{i}\right), \end{array}$$

with

(7) $$\begin{array}{c} S∼N_{n}\left(D\beta ,\Sigma \right), \end{array}$$

where the covariance matrix, Σ, of **S** is equal to the one defined for the Gaussian process (1). Conditioned in **S** the process {*Y*(**x**) : **x** ∈ *A* ⊂ *ℝ*^2^} consists of mutually independent random variables with *E*[*Y*_*i*_*|S*(**x**_*i*_)]=*μ*_*i*_. We have a known link function *g*(·) such that *g*(*μ*_*i*_)=*η*_*i*_; then, *μ*_*i*_=*g*^−1^(*η*_*i*_), *i*=1,…, *n*; in this case, the linear predictor is given by *η*_*i*_=*S*(**x**_*i*_); then, *μ*_*i*_=*g*^−1^(*S*(**x**_*i*_)). *D*=(**1**, **d**_1_,…, **d**_*p*_) is a known *n* × (*p*+1) design matrix, assumed of full-rank, with **1** a vector of *n* × 1 of ones and **d**_*j*_=(*d*_*j*_(**x**_1_),…,*d*_*j*_(**x**_*n*_))^*T*^, where *d*_*j*_(**x**_*i*_) is the value of the *j*-th covariate of the *i*-th location, and *β*=(*β*_0_, *β*_1_,…, *β*_*p*_) are the unknown regression parameters. In this model, the parameters to be estimated are *β*, *σ*^2^, and *ϕ* [28].

Markov Chain Monte Carlo (MCMC) algorithms are used in this work, for the computation of GLSM parameters within a Bayesian framework, which were provided by Diggle and colleagues at Lancaster University within the packages geoRglm [20] and geoR [21, 22], both of which are freely available within the framework of the open-source statistical system R Core Team [19]. We estimated each model using two chains of 500,000 iterations of MCMC sampling each, with a thinning of 100 samples and a burn of 50,000 samples. We computed the Potential Scale Reduction Factor (PSRF) (also called Rhat, $\hat{R}$) for each parameter [29]. Values $\hat{R}\leq 1.2$ were considered as proper convergence. Additionally, trace plots were examined. We calculate 95% credible intervals (CrI) for the coefficients of the models. If the intervals do not include zero, then they are considered to be statistically significant.

To rule out multicollinearity between the covariates of the model, the Pearson correlation coefficient was used [30].

#### 2.2.1. Spatial Autocorrelation

Moran's *I* statistic is an indicator of global spatial autocorrelation [31, 32], which is given by

(8) $$\begin{array}{c} I=\frac{n\sum_{i}^{n}\sum_{i}^{n}w_{ij}\left(y_{i}-\bar{y}\right)\left(y_{j}-\bar{y}\right)}{\sum_{i\neq j}^{n}w_{ij}\sum_{i}^{n}{\left(y_{i}-\bar{y}\right)}^{2}}, \end{array}$$

where *y*_*i*_ and *y*_*j*_ are detected cases of dengue in district *i* and *j*, respectively, $\bar{y}$ is the mean incidence over all the studied districts, and *n* is the total of localities. The proximity between district *i* and *j* is given by *w*_*ij*_; when locations *i* and *j* are neighbors, then *w*_*ij*_=1 and *w*_*ij*_=0; when they are not, for *i*=*j*, we take *w*_*ij*_=0. The weight, *w*_*ij*_, between district *i* and *j* is row-standardized (i.e., they sum to 1). In this analysis, the coefficient *I* was computed using adjacency (queen‘s case contiguity, that is, the district share edges and vertices). Municipalities do not have a neighbor *w*_*ij*_=0.

Local indicator of spatial association (LISA) is a decomposition of Moran's *I* and is used to identify the contribution of each location in the statistic [33]. LISA is given by

(9) $$\begin{array}{c} I_{i}=\frac{\left(y_{i}-\bar{y}\right)}{s^{2}}\sum_{j}w_{ij}\left(y_{j}-\bar{y}\right), \end{array}$$

where *s*^2^ is the global variance and the summation over *j* is such that only neighboring values *j* ∈ *J*_*i*_ are included. As for coefficient *I*, in cases where localities are used, *w*_*ij*_=1 for immediate neighbors of a locality and *w*_*ij*_=0 for all other localities. The coefficient *I*_*i*_ was used to derive significant spatial clustering through four cluster types: High-High, Low-Low, High-Low, and Low-High. For instance, the High-High cluster indicates localities with high values of a variable that are significantly surrounded by regions with similarly high values.

It is important to mention that the Moran and LISA index measures the degree of linear association of the values of the variable in neighboring regions. Thus, with these criteria, it will be possible to determine the existence of a spatial relationship in confirmed dengue cases.

To identify the spatial correlation between two variables and also to identify bivariate clustering, Bivariate Moran's *I* can be used [34]. The spatial correlation between two variables is an indication of the degree of linear association between the variable, *x*, and the variable *y* in neighboring regions ∑_*j*_*y*_*j*_*w*_*ij*_, but not in the same region. On the other hand, the bivariate grouping allows us to identify locations with high values in a first variable surrounded by locations with high values for a second variable (cluster High-High). The statistic is given by

(10) $$\begin{array}{c} I_{\text{Biv}}=\frac{n\sum_{i=1}^{n}\sum_{j=1}^{n}\left(x_{i}-\bar{x}\right)\left(y_{j}-\bar{y}\right)w_{ij}}{n_{b}\sum_{i=1}^{n}x_{i}^{2}}, \end{array}$$

where *n* is the number of regions, and *n*_*b*_ is the sum of the wight which simplifies to *n* if the spatial weight matrix is row-standardized, which for this study is what is done. The locations' variable for the area‘s proximity is given by *w*_*ij*_ which is the element from the corresponding spatial weight matrix.

#### 2.2.2. Identification of the Correlation Structure of the Gaussian Process

To determine a correlation function, from a family of parametric models, for the Gaussian process (7), the mean square normalized error criteria (MSNE) can be used:

(11) $$\begin{array}{c} \text{MSNE}=\frac{1}{n}\sum_{i=1}^{n}\frac{{\left(Y\left(x_{i}\right)-\hat{Y}\left(x_{i}\right)\right)}^{2}}{s^{2}\left(x_{i}\right)}, \end{array}$$

where *Y*(**x**_*i*_) is the observed value in *x*_*i*_ ∈ *A*, $\hat{Y}\left(x_{i}\right)$ is the predicted value in *x*_*i*_ ∈ *A*, and *s*^2^(**x**_*i*_) is the variance obtained from the interpolation method used [26]. For the interpolation of *Y*(**x**_*i*_), in this work, ordinary kriging was used. If the correlation model is correctly identified and well estimated for model (7), then the MSNE should be close to 1 [26].

#### 2.2.3. Spatial Model Validation

In spatial models to measure the accuracy and precision of the prediction (spatial interpolation), i.e., the error associated with the prediction, leave-one-out cross-validation can be used [35]. Among the statistics used to evaluate the prediction error are the mean absolute error (MAE)

(12) $$\begin{array}{c} \text{MAE}=\frac{1}{n}\sum_{i=1}^{n}\left|Y\left(x_{i}\right)-\hat{Y}\left(x_{i}\right)\right|, \end{array}$$

and the root mean square error (RMSE):

(13) $$\begin{array}{c} \text{RMSE}={\left[\frac{1}{n}\sum_{i=1}^{n}\left(Y\left(x_{i}\right)-\hat{Y}\left(x_{i}\right)\right)\right]}^{\left(1/2\right)}. \end{array}$$

According to Li and Heap [36], MAE is less sensitive than RMSE to the presence of extreme values. These statistics provide a measure of the size of the error associated with the interpolation method. Another way to evaluate the prediction of a spatial model is by means of the differences between the observed data and the predicted values at the sampled points. According to Li and Heap [36], the best statistics to evaluate the interpolation of a spatial model are MAE and RMSE. If the spatial interpolation is adequate, then MAE and RMSE should tend to zero. To evaluate the spatial model, the Pearson correlation coefficient of the observed data with the predicted values can also be calculated; if the spatial model is adequate, the correlation value tends to be 1. Leave-one-out cross-validation and Pearson's correlation coefficient were used to evaluate the spatial prediction of the model.

### 2.3. Prediction of Dengue Cases from the Developed Model

Confirmed DCs in the state of Chiapas can be seen as a counting variable that was observed in different locations. The GLSM (6) model allows us to study this type of variable in space, given a group of explanatory variables, because it takes into account the spatial dependency structure that exists in the realizations of the variable of interest. The selected explanatory variables will be used for the spatial prediction of DCs. To identify multicollinearity between the explanatory variables of the model, the *cor.test* function of the stats package [19] was used. For the spatial prediction of confirmed DCs, the *pois.krige.bayes* function of the geoRglm package [20] was used.

### 2.4. Bioethical Aspects

This study was approved (registration no. EADIS-17-2020) by the Ethics Committee at Health Secretary of State of Chiapas, and we obtained permission to access and use data from the database provided by the vectors section. The management of the database was regulated by the Official Mexican Standard NOM-024-SSA3-2012, where mention is made of the use and management of electronic medical records.

## 3. Results

In total, 573 confirmed cases of dengue were registered within the first 32 EWs in Chiapas, of which 49.04% occurred in Tuxtla Gutierrez (the state capital), the other cases happened in the remaining 35 municipalities, and most of them were female (53%). Meanwhile, 61.9% of DCs were registered in urban municipalities; 98% mentioned having the presence of the vector at home, 15% indicated that they did not have potable water, and 18% mentioned that they did not have a garbage collection service.

### 3.1. Georeferencing

**Figure 2:**
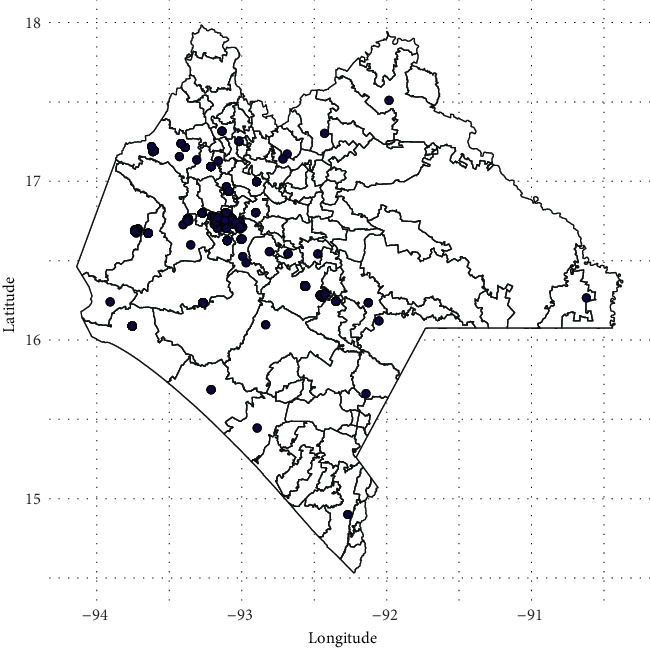
Spatial distributions of 573 cases of dengue confirmed in the state of Chiapas.

The spatial distribution of DCs in the state of Chiapas is heterogeneous. The 573 confirmed DCs located in the 36 municipalities of the state that corresponded to the analysis of DCs reported during the first 32 EWs of 2019 can be seen with blue dots in Figure 2.

**Figure 3:**
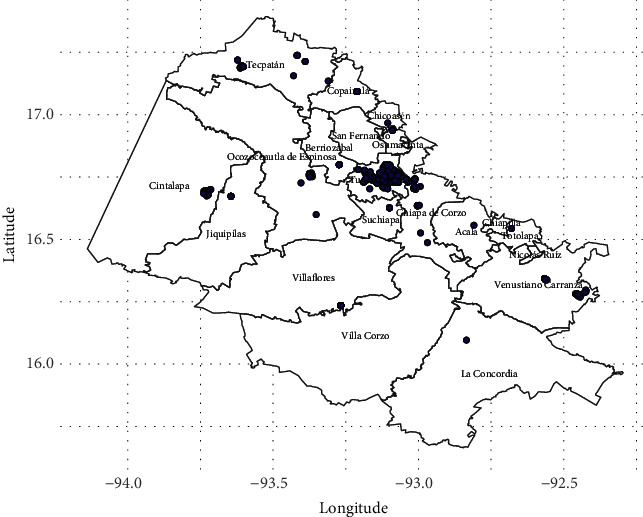
Municipalities of Chiapas with more confirmed cases of dengue.

The municipalities with the most confirmed dengue cases are located in western Chiapas (Figure 3), among them are Tuxtla Gutierrez (281 cases), Venustiano Carranza (47 cases), Chiapa de Corzo (38 cases), San Fernando (27, cases), Tecpatán (27 cases), Copainalá (21 cases), Ocozocoautla de Espinosa (19 cases), Cintalapa (16 cases), Osumacinta (11 cases), and Berriozáball (11 cases).

**Figure 4:**
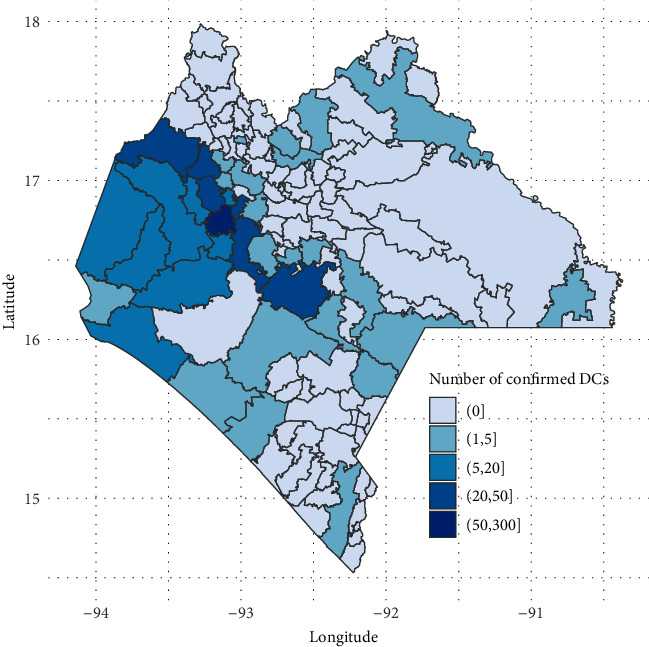
Municipalities of Chiapas with confirmed cases of dengue.

The distribution of the number of confirmed dengue cases by the municipality is shown in Figure 4. According to the graph, the municipalities with the highest number of cases are located in the west of Chiapas.

The capital of the state, together with the surrounding municipalities of Berriozábal, Chiapa de Corzo, and Suchiapa, generated the highest incidence of DCs (334 cases). Together, this area has the largest population in the state, with approximately 705,201 inhabitants. Meanwhile, the municipality of Venustiano Carranza had 47 cases of dengue, which is equivalent to 8.2% of the total report.

### 3.2. Model Variables

Table 1 shows that the mean maximum temperature of confirmed DCs was 33.7 (SD 4.011) °C. The mean altitude was 600.6 (SD 197.52) meters, with a range of 501 to 600 meters above sea level. The mean rainfall was 214.9 (SD 35.89) mm, and most DCs occurred in the rainy season. The age of the confirmed DCs was 14.37 (SD 11.15) years. Most of the cases occurred between 5 and 9 years, and there was a case of a newborn with dengue infection (Age = 0).

### 3.3. Spatial Correlation

We calculated the spatial correlation of the numbers of confirmed DCs and the municipalities. The value of the Moran index is 0.115, which indicates that there is a spatial association in the number of confirmed DCs in the Chiapas municipalities (*p* value=0.001).

**Figure 5:**
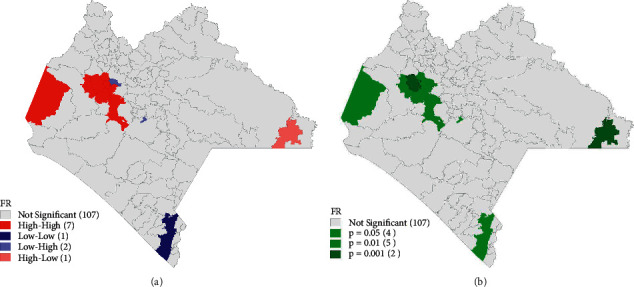
The local spatial autocorrelation indicators (LISAs). (a) Clusters of risk. (b)*p* value.

We observed significant spatial autocorrelation (Moran's *I* = 0.218, *p*-valor = 0.002), indicating that confirmed Dc‘s rates between municipalities are significantly spatially related. The LISAs show the heat and significance maps corresponding to significant clusters (Figure 5(a)); a High-High group (red color, Figure 5(b)) of 7 municipalities was identified: Chicoasen, Cintalapa, Berriozábal, San Fernando, Tuxtla Gutierrez, Osumacinta, and Chiapa de Corzo; these municipalities present rates (FR : number of confirmed cases of dengue between total population of the municipality) of significant dengue (Figure 5(b)) and high surrounded by municipalities with equally high rates. The second important group is Low-High (light blue Figure 5(b)) composed of the municipalities Nicolás Ruíz and Soyalo, which are municipalities with significantly low rates despite being surrounded by municipalities with higher rates.

Rates of confirmed DCs by municipality: Number of confirmed cases of dengue among total population of the municipality.

### 3.4. Evaluation of the Model Structure

Assuming a Matérn correlation function (5) for the spatial process (7), we have MSNE=0.970, which implies that the Matérn correlation function explains the spatial correlation structure of confirmed dengue cases. DCs will be considered as a dependent variable, and maximum temperature, altitude of the municipality, and rainfall are considered covariables for the spatial process. Pearson's correlation coefficient shows that there is no multicollinearity problem in the covariates of the model: altitude and maximum temperature (*r*=−0.2231, *p* value=0.1909), altitude and rainfall (*r*=−0.07685, *p* value=0.656), and finally rainfall and maximum temperature (*r*=−0.14124, *p* value=0.4113).

**Figure 6:**
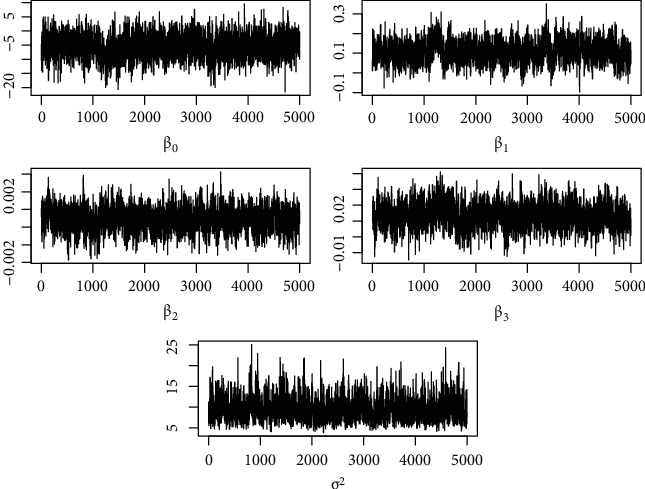
Time series plots showing the MCMC output every 10-th iteration.

**Figure 7:**
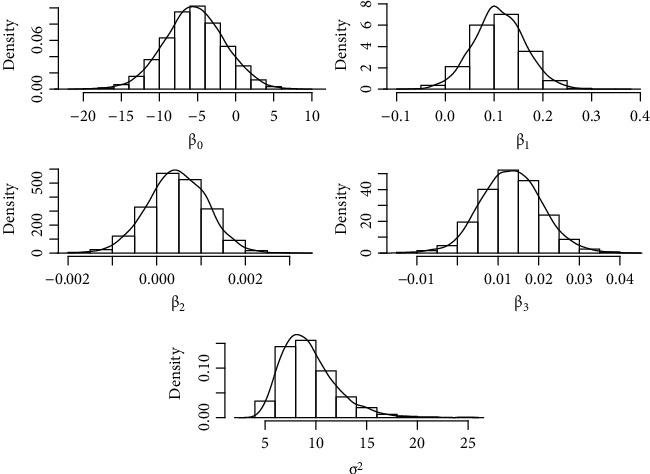
Posterior distribution of the model parameters.

We show the results of the Bayesian inference implemented via MCMC algorithms. Each trace consists of 4500 values sampled from the posterior distributions of *σ*^2^ and *β*. As shown in Table 2, the MCMC converged because all the $\hat{R}$ were less than 1.2 and the trace plots of Figure 6 show that the two chains mix well.  Figure 7 shows the histograms of the empirical posterior distributions of the parameters *β*_*j*_. These posterior distributions are approximately Gaussian, and for the parameter *σ*^2^ which was inverse Chi-squared, we set *ϕ*=200.

Table 3 shows the posterior mean, median, and 95% credible interval for each of the parameters of GLSM. The confirmed cases of dengue in Chiapas increase with maximum temperature, altitude, and rainfall. Age does not significantly affect the disease because the 95% posterior interval of *β*_4_ contains zero.

Given that age is not significant, it is removed from the model and adjusted again (see Table 4).

### 3.5. Prediction of the Model to the Chiapas Map

The error associated with spatial prediction, using leave-one-out cross-validation, was MAE=14.84309 and RMSE=3.48654, which are small values considering that the sample size available is 36 municipalities. Pearson's correlation coefficient of the observed and predicted values was 0.318, which is significant at *α*=0.10. These results give evidence that the fitted model performs good spatial prediction.

**Figure 8:**
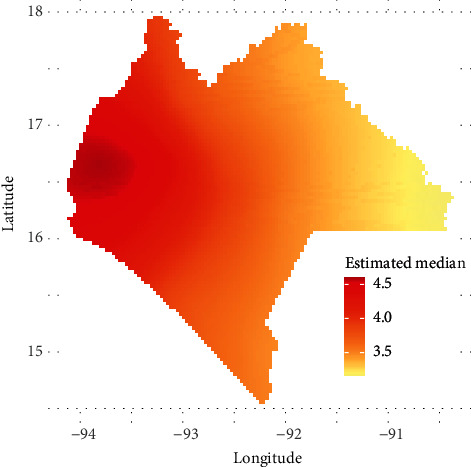
Interpolation of confirmed cases of dengue.

The prediction of the model on Chiapas maps was carried out based on the places where the cases were registered. As can be seen in Figure 8, the prediction is displayed on a gradient map from yellow to red, which refers to the estimated median. The prediction power is notably better in the regions that have a greater number of municipalities where DCs are registered. Outside of these areas, the prediction power is considerably diminished.

For predicting, the variables that were significant and that affect the number of confirmed DCs (e.g., rainfall, maximum temperature, and altitude of each municipality) were taken.

**Figure 9:**
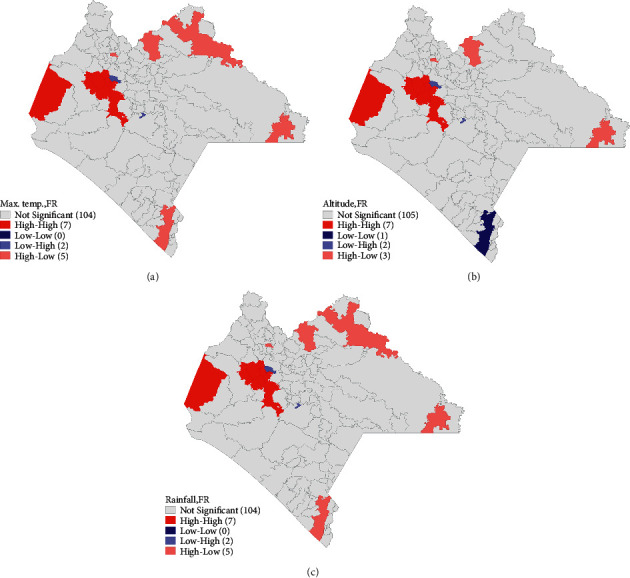
Significant spatial clustering of bivariate LISA for each of the significant variables in the spatial model and rate of confirmed dengue cases. Clusters of risk are shown.

Bivariate cluster maps were needed for each of the significant variables in an estimated spatial model, in order to better understand the individual spatial effect of each of the covariates on confirmed DCs rates. We observed a positive spatial relationship between maximum temperature and rates of confirmed DCs (*I*_Biv_=0.271, *p* value=0.001); municipalities with high temperatures are surrounded by municipalities with high confirmed DCs rates (Figure 9(a)). There is also a positive spatial relationship between altitude and confirmed DCs rates (*I*_Biv_=0.199, *p* value=0.001); municipalities with high altitudes are surrounded by municipalities with high confirmed DC rates (Figure 9(b)). Finally, we also observe a positive spatial relationship between rainfall and confirmed DC rates (*I*_Biv_=0.238, *p* value=0.001); municipalities with high rainfall are surrounded by municipalities with high rates of confirmed DCs (Figure 9(c)).

## 4. Discussion

Of the total registered DCs (*n* = 573), the 49.04% occurred in Tuxtla Gutierrez (the state capital) and most of them were female (53%); also, the capital of the state together with the surrounding municipalities (Berriozábal, Chiapa de Corzo and Suchiapa) generated the highest incidence of dengue cases (*n* = 334), which also together have the largest population in the state, with approximately 705,201 inhabitants. Further, 15% of localities analyzed indicated that they did not have potable water and 18% mentioned that they did not have garbage collection services. Our observations are consistent with a population potentially influenced by demographic changes, such as the birthplace and socioeconomic data [37] with similar lifestyles and social interactions between neighboring areas since human mobility is also an important factor in the dynamics of dengue transmission [37–41]. Therefore, the results presented here could be generalized to similar epidemiological settings.

On the other hand, in confirmed dengue cases, we observed that the average age was 14 years. This result was similar to that obtained by Phanitchat [5] and collaborators, who reported in northeast Thailand that the age range of dengue cases was between 5 and 14 predominating 15 years old.

Different studies have shown how environmental, sociodemographic, geographical, and entomological factors play a significant role in dengue infection, with climatic conditions being the most associated with the disease because of their relationship with the vector [5, 6, 11, 13, 14, 40].

Statistical models have been used for the prediction of DCs in endemic areas to analyze the relationship of risk factors; it has been observed that the combination of climatological, sociodemographic, entomological, and geographical variables is explanatory of DCs [6, 41]. Due to the areas of vulnerability in the state of Chiapas, it is necessary to establish control strategies to prevent new cases of dengue; for this reason, we propose predicting the spatial dispersion of DCs in Chiapas based on the climatological and nonclimatological variables of the region. In the current study, the climatological variables rainfall and maximum temperature and the geographical variable altitude were considered, in order to be able to make a prediction map, using GIS since they are tools that facilitate analysis and favor the visualization of data and results as mentioned by Mala and Jat [40].

Regarding the temperature variable, the maximum recorded of confirmed DCs was 33.7°C (SD 4.011), while the mean rainfall was 214.9 mm (SD 35.89), observing that the most DCs occurred in the rainy season. With those data, we can reinforce what was observed by Phanitchat regarding the fact that climatological variables have an effect on dengue infection [5]. They mention that there were clear seasonal patterns of dengue incidence during the rainy seasons, thus showing that rainfall also has significant effects on dengue transmission, findings that also coincide with the study by Wangdi et al. [42], and another correlational study that analyzed the rainfall and clinical dengue cases also found that the dengue incidence was closely related to rainfall [43]. In addition, temperature is another primary environmental risk factor for dengue transmission; higher temperatures enhance viral replication in the vector mosquito in a shorter amount of time and thus increase the transmission potential of dengue viruses. In this way, a study of the extrinsic incubation period found that the virus remained in the midgut at 18°C but could disseminate and invade the salivary glands at temperatures between 23°C and 32°C [44], thereby showing higher temperatures produce a shorter extrinsic incubation period and greater transmission potential.

For these reasons, the strong relationships between rainfall and temperature with the number of DCs have been used to develop prediction models [45, 46]. For our study, rainfall and temperature turned out to be significant variables for the prediction model, discarding the other climatological variables due to the absence of data in the climatic information records for our study, highlighting that rainfall and temperature are environmental risk factors for dengue transmission.

Interestingly, the geographical variable altitude was 600.6 meters (SD 197.52), with a range of 501 to 600 meters above sea level, but turned out to be a significant variable for the study, which coincides with the results of the work of Aswi and collaborators [6], where this variable was used in different statistical models in order to describe the behavior of the disease since the spread of the *Aedes aegypti* mosquitoes is limited by climatic conditions, and this will be governed by the location of the geographical area and its altitude. Reinhold et al. [47] mention that the *Aedes aegypti* mosquito is an endothermic arthropod that cannot regulate its body temperature and therefore this is defined by the climatic conditions of the environment, so the geographical location and altitude are important variables for dengue disease.

The relationships between variables maximum temperature, altitude of the municipality, and rainfall with DCs allowed us to generate the LISA that shows the heat and significance maps corresponding to significant clusters (the High-High group with 7 municipalities and Low-High with 2 municipalities), noting the importance of these variables, to prevent future cases of dengue in these vulnerable areas.

Although our study has some limitations, it is important to mention that this ecological study and only RT-PCR confirmed cases were considered. In addition, the information on climatological variables was scarce for some municipalities due to the fact that the meteorological stations have faults and this led to the fact that important climatological variables such as wind speed and humidity were not considered. Furthermore, other demographic variables like human movement and entomological variables were not considered.

## 5. Conclusion

It was found that altitude, rainfall, and maximum temperature were the factors that best predicted the presence of DCs in the state of Chiapas in the period from January to August 2019. This model took into account climatological and geographical variables of the region to predict DCs in the state of Chiapas, and it offers observation of the prediction of the model with a map. Therefore, the model shows the importance of taking into account the before mentioned variables to prevent future cases of dengue in these vulnerable areas.

## Figures and Tables

**Table 1:** Descriptive statistics of covariables.

Statistic	Maximum temp. (°C)	Altitude (meters)	Rainfall (mm)	Age (years)
Mean	33.75	600.6	214.9	14.37
Median	34.9	600.0	220.9	11.0
Minimum	17.0	50.0	130.9	0.0
Maximum	41.3	1600.0	267.0	70.0
Standard deviation (SD)	4.011	197.52	35.89	11.15

**Table 2:** Gelman and Rubin's convergence diagnostic to the parameter.

Parameter	$\hat{R}$	Upper CrI
Intercept (*β*_0_)	1.00	1.01
Maximum temperature (*β*_1_)	1.00	1.02
Altitude (*β*_2_)	1.00	1.00
Rainfall (*β*_3_)	1.00	1.02

**Table 3:** Credible intervals of the intercept and covariates of the saturated model.

Parameter	Mean	Median	95% credible intervals
Intercept (*β*_0_)	−3.00891	−3.09526	−5.79748, 5.29488
Maximum temp. (*β*_1_)	0.08744	0.08707	0.05190, 0.19173
Altitude (*β*_2_)	0.00056	0.00056	0.00013, 0.00185
Rainfall (*β*_3_)	0.01037	0.01020	0.00525, 0.02544
Age (*β*_4_)	−0.05379	−0.05488	−0.08104, 0.02541
*σ* ^2^	9.03115	8.56717	7.09705, 14.21415

**Table 4:** Credible intervals of the intercept and covariates of the selected model.

Parameter	Mean	Median	95% credible intervals
Intercept (*β*_0_)	−5.32606	−5.40243	−7.97215, 2.38377
Maximum temp. (*β*_1_)	0.11063	0.10896	0.07607, 0.21557
Altitude (*β*_2_)	0.00045	0.00046	0.00002, 0.00174
Rainfall (*β*_3_)	0.01327	0.01327	0.00828, 0.02830
*σ* ^2^	9.29359	8.87915	7.39459, 14.39212

## Data Availability

All relevant study data are provided within the article.

## Abbreviations

CrI: Credible intervals
DCs: Dengue cases
DENV: Dengue virus
EWs: Epidemiological weeks
FR: Number of confirmed cases of dengue in the total population of the municipality
GIS: Geographic Information System
GLSM: Generalized linear spatial model
INEGI: National Institute of Statistics and Geography
LISA: Local indicator of spatial association
MCMC: Markov Chain Monte Carlo
MSNE: Mean square normalized error
RT-PCR: Reverse transcription-polymerase chain reaction
SD: Standard deviation
WHO: World Health Organization
WMO: World Meteorological Organization
WGS84: World Geodetic System.
